# Future interactions in Particle Toxicology: the role of *PFT*

**DOI:** 10.1186/1743-8977-5-5

**Published:** 2008-04-07

**Authors:** Paul JA Borm

**Affiliations:** 1 Zuyd University, Heerlen, The Netherlands

Particle research and particle toxicology have been historically closely connected to industrial activities or materials, such as coal, asbestos and manmade mineral fibers. Until the early nineties papers on these icons of exposure dominated the literature in classical journals with a strong occupational or hygiene approach. Along with the evolution in our understanding of general disease, particle toxicology has benefited from the developments in molecular medicine. Over time there has been a considerable change in the experimental approach taken by particle toxicologists. Nowadays inflammation as a key response to particle deposition in tissue and a process in particle effects has been inexorable and inflammation now lies at the very heart of our understanding of lung and systemic disease associated with particle exposure. As in all molecular medicine, there has been a huge concentration on the regulation of gene expression as a basis for disease, and this continues. Oxidative stress has emerged as the dominant paradigm for how particles initiate inflammation and genotoxicity, a stress further augmented by inflammatory cells releasing their arsenal of oxidants.

Between 1995 and 2005, the health effects of ambient particulate matter and explanations for their effects have dominated research meetings and journals. Ambient particles suddenly were responsible for effects not only in the lung, but also in the heart, the vascular system and recently also the brain [1,2]. A new age of interaction with other disciplines and clinical specialties was started. With these changing targets and approaches also the array of journals available for publication expanded, and interestingly particle-induced effects have reached the forefront of the general medical journals, as well the specialty journals on lung, heart and other diseases. Particle research has reached a professional quality and impact it has never seen before.

*Particle and Fibre Toxicology *was started in 2004 at the very summit of this interaction, and has published articles on a variety of toxicological endpoints and mechanisms associated to particles. A total of 45 was articles were published in four years, on a variety of particles. About half of the articles covered particles such as asbestos, quartz, diesel or ambient particle matter. Although the number of manuscripts was rather low over the total period, the citation data based on Scopus data [3] showed 250 cites on the first 40 articles. Based on this data, if the journal was to receive an impact factor for 2007 (based on 2007 citations to 2005 and 2006 articles) this would be in the region of 5.7. The high number of citations is in part driven by a number of review articles concerning the effects of nanomaterials and combustion nanoparticles [2,4-6]. In addition, the fact that *Particle and Fibre Toxicology *is the only journal on this topic which is free access and online, has contributed extensively on its use and citation in many other journals and reports.

The journal wants to play a decisive role in a decade where particle research will be challenged and driven by the developments and applications of nanomaterials. Already more than 40% of articles in *Particle and Fibre Toxicology *since the start of the journal in late 2004 were on this topic, and we want to stimulate the interaction of producers, users and testers of nanomaterials in a variety of fields. They are used in new polymers, so-called nanocomposites and coatings, but also in drug delivery as well as medical imaging through MRI and fluorescence. Most of these applications and particles are new to the field, and we have only started to understand how surface and its chemical properties do determine their aptness for interaction with biological targets. However, studies have demonstrated that even inert materials like gold and TiO2 in nanosize can become chemically active. In other words, inert materials can become reactive just by making them smaller.

Nowadays nanoparticles are generated from highly skilled research labs and produced for many applications and in many countries. In the near future upscaling of production is expected and worker exposure and consumer contact are expected to increase dramatically over the years to come. Nanomaterials have already proven to be slippery customers for regulators and regulations such as REACH, since one chemical identity can show different physical properties based in size and shape. Consequently the field of particle toxicology faces a number of challenges and opportunities in the near future. These challenges include:

- to generate understanding of the biological action of nanomaterials

- denominate the relevant material metrics

- attract new young top researchers to the field

- Use, communicate and adapt current particle paradigms

- Bridge know-how between different application area's of nanomaterials

- Communicate findings to the broad public in an increasing relevant society

These challenges imposed by nanomaterials demand for new collaborations between toxicologist and chemists, material scientists, and engineers. Competence centers, or virtual networks, where nanomaterials can be made and tested, will be vital for further development of sustainable nanomaterials. *Particle and Fibre Toxicology *wants to play a role in this process, and has recently recruited three new Associate Editors from China, Japan and the USA and is still working on a complete Editorial Board setting. In addition, we now welcome manuscripts on areas such as *biomaterials, drug delivery and imaging *in an effort to bridge the available know-how in classic particle toxicology to these areas that have co-existed without much communication for so long.

As the new Editor-in-Chief of this journal I firmly believe in open access publication. I also find it a privilege to contribute to the development of particle toxicology as an open access discipline for many other disciplines, in their effort to understand the toxicological action of (nano) materials, thereby creating a platform to help and create sustainable materials for the future.
